# *ERBB2* mutations define a subgroup of endometrial carcinomas associated with high tumor mutational burden and the microsatellite instability‐high (MSI‐H) molecular subtype

**DOI:** 10.1002/1878-0261.13698

**Published:** 2024-07-19

**Authors:** Melica Nourmoussavi Brodeur, Pier Selenica, Weining Ma, Sara Moufarrij, Christian Dagher, Thais Basili, Nadeem R. Abu‐Rustum, Carol Aghajanian, Qin Zhou, Alexia Iasonos, Lora H. Ellenson, Britta Weigelt, M. Herman Chui

**Affiliations:** ^1^ Department of Pathology and Laboratory Medicine Memorial Sloan Kettering Cancer Center New York NY USA; ^2^ Department of Radiology Memorial Sloan Kettering Cancer Center New York NY USA; ^3^ Department of Surgery Memorial Sloan Kettering Cancer Center New York NY USA; ^4^ Department of Medicine Memorial Sloan Kettering Cancer Center New York NY USA; ^5^ Department of Epidemiology and Biostatistics Memorial Sloan Kettering Cancer Center New York NY USA; ^6^ Present address: Department of Obstetrics & Gynecology McGill University Montreal Canada

**Keywords:** endometrial cancer, ERBB2, HER2, microsatellite instability, mutation

## Abstract

Anti‐HER2 therapy is indicated for erb‐b2 receptor tyrosine kinase 2 (*ERBB2*)‐amplified/overexpressing endometrial carcinoma (EC). Mutations constitute another mode of *ERBB2* activation, but only rare *ERBB2*‐mutated ECs have been reported. We sought to characterize the clinicopathologic and genetic features of *ERBB2*‐mutated EC. From an institutional cohort of 2638 ECs subjected to clinical tumor‐normal panel sequencing, 69 (2.6%) with pathogenic *ERBB2* mutation(s) were identified, of which 11 were also *ERBB2*‐amplified. The most frequent *ERBB2* hotspot mutations were V842I (38%) and R678Q (25%). *ERBB2* mutations were clonal in 87% of evaluable cases. Immunohistochemistry revealed low HER2 protein expression in most *ERBB2*‐mutated ECs (0/1+ in 66%, 2+ in 27%); all 3+ tumors (7.3%) were also *ERBB2*‐amplified. Compared to *ERBB2*‐wildtype ECs (with or without *ERBB2* amplification), *ERBB2*‐mutated/non‐amplified ECs were enriched for the microsatellite instability‐high (MSI‐H) and, to a lesser extent, DNA polymerase epsilon, catalytic subunit (*POLE*) molecular subtypes, and associated with high tumor mutational burden and low chromosomal instability. Survival outcomes were similar between patients with *ERBB2*‐mutated/non‐amplified versus wildtype EC, whereas *ERBB2* amplification was associated with worse prognosis on univariate, but not multivariate, analyses. In conclusion, *ERBB2* mutation defines a rare subgroup of ECs that is pathogenically distinct from *ERBB2*‐wildtype and *ERBB2*‐amplified ECs.

## Graphical abstract

*ERBB2* amplification is a known driver of copy number‐high/*TP53*‐abnormal high‐grade endometrial carcinoma and is associated with HER2 overexpression. In this retrospective cohort of clinically annotated endometrial carcinomas, *ERBB2* pathogenic mutations define a distinct and non‐overlapping subgroup of tumors, typically with low levels of HER2 expression, high tumor mutational burden, microsatellite instability‐high status, and low‐grade endometrioid histology.

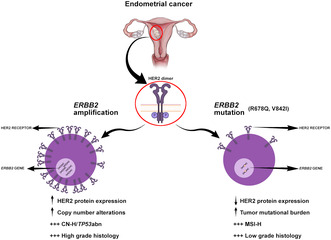

**Abbreviations**
ADC: antibody‐drug conjugate
amp: amplified
CI: confidence interval
CN‐H: copy number‐high
CN‐L: copy number‐low
EC: endometrial carcinoma
*ERBB2*
: erb‐b2 receptor tyrosine kinase 2
FGA: fraction of genome altered
FIGO: International Federation of Gynecology and Obstetrics
HR: hazard ratio
IHC: immunohistochemistry
IQR: interquartile range
MMR: mismatch repair
MSI‐H: microsatellite instability‐high
MSK‐IMPACT: Memorial Sloan Kettering‐Integrated Mutation Profiling of Actionable Cancer Targets
mut: mutated
non‐amp: non‐amplified
NOS: not otherwise specified
NSMP: no specific molecular profile
OS: overall survival
PFS: progression‐free survival
*POLE*
: DNA polymerase epsilon, catalytic subunit
SNV: single nucleotide variant
TCGA: The Cancer Genome Atlas
TMB: tumor mutational burden
*TP53*abn: *TP53* abnormal
wt: wildtype

## 1 Introduction

Endometrial carcinoma (EC) is the most common gynecologic malignancy, with endometrioid, serous, and clear cell carcinomas comprising the major histologic subtypes. Complementing the traditional histologic classification, The Cancer Genome Atlas (TCGA) study of EC identified four molecular subtypes [1]: (1) DNA polymerase epsilon, catalytic subunit (*POLE*), ultra‐mutated; (2) microsatellite instability‐high (MSI‐H), hypermutated; (3) copy number‐high (CN‐H), serous‐like; and (4) copy number‐low (CN‐L), endometrioid. These molecular subtype classes are associated with distinct outcomes, with *POLE* having the most favorable outcome, MSI‐H and CN‐L intermediate outcomes, and CN‐H ECs having the worst outcomes.

There is recent interest in the tyrosine kinase receptor HER2, encoded by the erb‐b2 receptor tyrosine kinase 2 (*ERBB2*) oncogene, as a therapeutic target for high‐grade ECs. *ERBB2* amplification leads to HER2 protein overexpression and is correlated with poor prognosis in several tumor types, including breast, gastroesophageal, and ECs [2, 3, 4]. Anti‐HER2 therapies, including the monoclonal antibody, trastuzumab, constitute an important therapeutic option for HER2‐positive breast and gastroesophageal tumors [5]. Trastuzumab has been incorporated into National Comprehensive Cancer Network guidelines for treatment of advanced and recurrent serous EC with HER2 overexpression/*ERBB2* amplification [6], based on a randomized phase II study demonstrating improved survival outcomes in this patient population [7, 8].

*ERBB2* amplification in EC is primarily restricted to those of CN‐H/*TP53*‐abnormal (*TP53*abn) molecular subtype [9, 10], and *ERBB2*‐amplified serous/high‐grade carcinomas likely represent only a subset of all HER2‐driven ECs. In addition to amplification, *ERBB2* may also be altered by somatic mutations, which has been described in other tumor types, including breast, bladder, gastrointestinal, and lung cancers [11, 12, 13, 14, 15]. Pan‐cancer sequencing studies have revealed *ERBB2* mutations to be most common in bladder/urinary tract cancers (7–8%), followed by stomach (4–5%) and bile duct (4–5%) cancers [11, 12, 13, 14, 15]. Mutations involving the tyrosine kinase domain, encompassing exons 19, 20, and 21 (amino acids 720–987) are most prevalent overall, however, specific mutations vary in frequency between different tumor types. For example, in non‐small cell lung cancer, the most common mutation is p.Y772_A775dup, while biliary tract and breast cancers more commonly harbor S310F/Y and L755 mutations, respectively [11, 12].

*In vitro* overexpression systems have shown most mutations ultimately increase kinase activity, resulting in HER2 phosphorylation and activation of downstream signaling, accompanied by cellular transformation [11, 16]. *ERBB2* mutations are generally considered to confer resistance to trastuzumab, through constitutive activation of kinase activity, despite receptor blockade, or by interfering with drug binding [17]. However, neratinib, an irreversible pan‐HER tyrosine kinase inhibitor, demonstrated promising pre‐clinical activity across different types of *ERBB2* mutations, which led to a Phase II basket trial, SUMMIT (NCT01953926), evaluating neratinib in advanced pre‐treated *ERBB2*‐mutant solid tumors [18]. Clinical responses were variable and dependent on cancer type (with clinical efficacy observed primarily in breast, biliary and cervical cancers), the specific *ERBB2* mutation, and presence of other co‐existing mutations. New opportunities for targeting *ERBB2* mutations have also emerged with the development of antibody‐drug conjugates (ADCs), including trastuzumab emtansine [19, 20] and trastuzumab deruxtecan [21], which have shown clinical activity in patients with non‐small cell lung cancer with *ERBB2* mutations.

Unlike other cancer types, the prevalence and spectrum of *ERBB2* mutations in EC, as well as their clinicopathologic associations have not been well characterized. This knowledge could potentially pave the way towards exploring novel therapies to target *ERBB2* mutations in this tumor type. Therefore, in this study, we sought to characterize the clinical, histopathologic and genetic features of ECs harboring pathogenic *ERBB2* mutations.

## 2 Materials and methods

### 2.1 Case selection

The study methodology conforms to the standards set by the Declaration of Helsinki. The study was approved by Memorial Sloan Kettering Cancer Center Institutional Review Board and written informed consent for molecular profiling was obtained from all patients (IRB #12‐245). Of consented EC patients who underwent clinical FDA‐authorized tumor‐normal sequencing using Memorial Sloan Kettering‐Integrated Mutation Profiling of Actionable Cancer Targets (MSK‐IMPACT) [22], between 1/2014 and 03/2022 (*n* = 2638), ECs with pathogenic *ERBB2* mutations were identified [23]. Demographic and clinicopathologic data, including age at diagnosis, International Federation of Gynecologic and Obstetrics (FIGO) 2009 stage, clinical follow‐up, as well as information on anti‐HER2 therapy and radiologic response, if applicable, were extracted from electronic medical records. For comparison, 1790 *ERBB2* wildtype ECs (including those with *ERBB2* amplification, *n* = 99), annotated with clinical and molecular subtype information (see Section 2.3), were identified from a previously published dataset (1/2014–12/2020) [24]. For analysis of data from the Cancer Genome Atlas (TCGA) study of EC [1], information on *ERBB2* mutation, tumor histology and molecular subtype were extracted from the cBioPortal for Cancer Genomics website (http://www.cbioportal.org).

### 2.2 Sequencing analysis

All ECs included underwent clinical FDA‐authorized tumor‐normal MSK‐IMPACT panel sequencing targeting 341–505 genes, as previously reported [25]. Somatic mutations and tumor mutational burden were extracted from MSK‐IMPACT. *ERBB2* somatic mutations were considered pathogenic based on OncoKB [23]. Copy number alterations and loss of heterozygosity (LOH) were defined using facets [26], as previously described [27, 28]. The cancer cell fractions of somatic mutations were computed using absolute (v1.0.6) [29], and a mutation was classified as clonal if its probability of being clonal was > 50% or if the lower bound of the 95% confidence interval of its cancer cell fraction was > 90%, as previously described [27, 28].

### 2.3 EC molecular subtype classification

Molecular subtyping was performed using our previously described integrated molecular – immunohistochemistry (IHC)‐based approach [24]. In brief, ECs were classified as (1) *POLE* molecular subtype based on the presence of a *POLE* hotspot exonuclease domain mutation [30], (2) MSI‐H molecular subtype if the MSK‐IMPACT‐based MSIsensor score [31] was ≥ 10 and/or DNA mismatch repair‐deficient (MMR)‐deficient based on IHC, (3) CN‐H/*TP53*abn molecular subtype based on the presence of a pathogenic *TP53* genetic alteration, or (4) CN‐L/no specific molecular profile (NSMP) if any of the defining features of the other subtypes were lacking.

### 2.4 Histopathologic review and immunohistochemical analysis

All available diagnostic slides from *ERBB2*‐mutated (*ERBB2*‐mut) ECs were re‐reviewed by a gynecologic pathologist (M.H.C.) for confirmation of histological subtype and grade, according to WHO 2020 criteria [32]. HER2 IHC was performed (clone 4B5; Ventana, Tucson, AZ, USA) on all available cases, on the same tissue block used for MSK‐IMPACT sequencing. The percentage of tumor cells with absent, weak, moderate, or strong membranous staining was estimated, and HER2 IHC score was assigned, using newly proposed EC‐specific guidelines, based on criteria used in the clinical trial by Fader et al. [7, 8], endorsed by the College of American Pathologists [33].

### 2.5 Statistical analysis

Correlative analyses between *ERBB2* mutation status and clinicopathologic variables were performed using Wilcoxon rank sum test and Fisher's exact test, for continuous and categorical variables, respectively, with multiple comparisons adjusted using the Benjamini and Hochberg method. For survival analyses, only patients who received their primary treatment at MSK and had MSK‐IMPACT sequencing performed on primary tumors were included [*n* = 1012, including *ERBB2*‐mut/non‐amplified (mut/non‐amp), *n* = 34, *ERBB2*‐wildtype/non‐amplified (wt/non‐amp), *n* = 936, *ERBB2*‐wildtype/amplified (wt/amp), *n* = 39, *ERBB2*‐mut/amplified (mut/amp), *n* = 3]. Progression‐free survival (PFS) was defined from the time of pathologic diagnosis of EC to first recurrence or progression, by imaging or pathologic confirmation, death or last follow‐up date, whichever came first. Overall survival (OS) was defined as the time from diagnosis to death or last follow‐up. Non‐events were censored at the last follow‐up date. Left truncation methodology was applied to address selection bias as patients needed to be selected after the date of MSK‐IMPACT, as previously described [34]. Survival curves were generated using the Kaplan–Meier survival method, and hazard ratios (HR) with 95% confidence intervals (CI) and *P*‐values were obtained by the Cox proportional hazard model, accounting for left truncation. All tests were two‐sided and a *P*‐value < 0.05 was considered statistically significant. All analyses were performed using r version 4.1.2 (https://www.R‐project.org/).

## 3 Results

### 3.1 ERBB2 mutations in EC

**Fig. 1:**
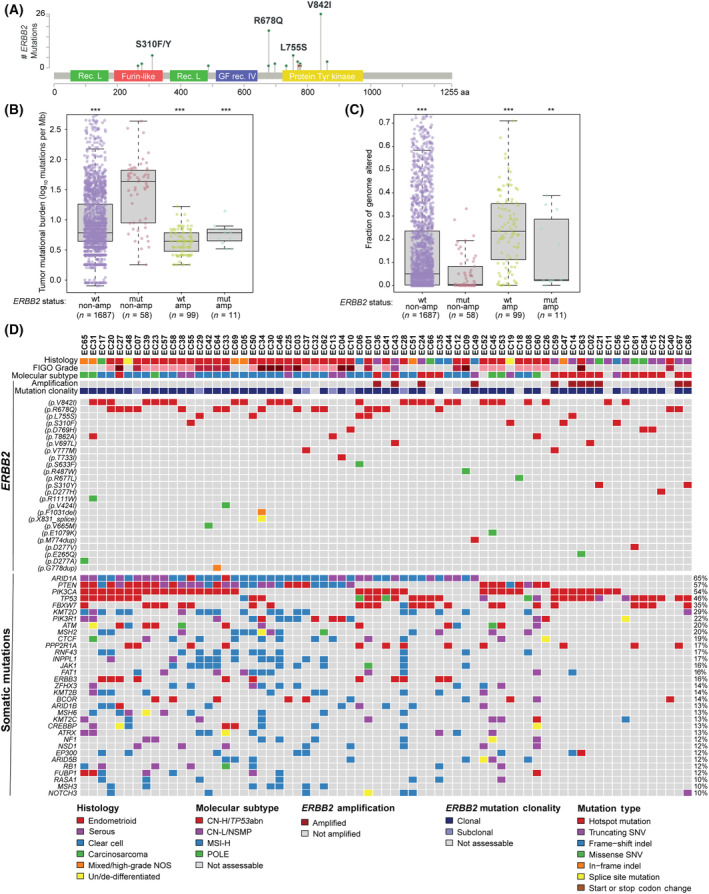
Genomic landscape of 69 *ERBB2*‐mutated endometrial carcinomas. (A) Lollipop plot showing frequencies of specific *ERBB2* activating mutations. (B, C) Targeted panel sequencing‐based tumor mutational burden (B) and fraction of genome altered (C), stratified by *ERBB2* mutation (wt, wildtype; mut, mutated) and copy number (non‐amp, non‐amplified; amp, amplified) status. Boxplots show median with interquartile range (IQR), with boundaries of whiskers at 1.5 times IQR. **P < 0.01, ***P < 0.001, Wilcoxon rank sum test. (D) Oncoplot displaying *ERBB2* mutations and recurrent somatic mutations in *ERBB2*‐mutated endometrial cancers. Mutation types, histologic subtype, molecular subtype, *ERBB2* amplification status, and clonality of *ERBB2* mutations are annotated according to the legend. CN‐H/*TP53*abn, copy number‐high/*TP53* abnormal; CN‐L/NSMP, copy number‐low/no specific molecular profile; high‐grade EC‐NOS, high‐grade endometrial carcinoma, not otherwise specified; Indel, insertion/deletion; MSI‐H, microsatellite instability‐high; SNV, single nucleotide variant.

From a cohort of 2638 ECs across histologic types, 69 (2.6%) had known pathogenic *ERBB2* mutations, of which 11 (16%) also had concurrent *ERBB2* amplification, and 8 (12%) ECs harbored multiple pathogenic *ERBB2* mutations. The most frequent *ERBB2* hotspot mutations were V842I (26/69, 38%), located in the kinase domain, and R678Q (16/69, 23%), situated in the juxtamembrane domain (Fig. 1A). Other recurrent mutations included kinase domain mutations (L755S, *n* = 5; D769H, *n* = 3; T862A, *n* = 3; V777M, *n* = 2), and mutations involving the furin‐like extracellular domain (S310F/Y, *n* = 6) and juxtamembrane domain (V697L, *n* = 2). Of 61 evaluable cases with sufficient tumor purity, assessment of the cancer cell fractions revealed that *ERBB2* mutations were clonal in 87% (*n* = 53) of cases.

### 3.2 Somatic genetic landscape of ERBB2‐mutated ECs

The global genomic landscape of *ERBB2*‐mut/non‐amp ECs was characterized by a significantly higher tumor mutational burden (TMB; median 43.2 mutations per Mb, range: 1.8–436.2), relative to ECs lacking *ERBB2* mutation or amplification (*ERBB2*‐wt/non‐amp: median 6.1 mutations per Mb, range: 0.8–667.9, *P* < 0.001) and ECs with *ERBB2* amplification, but no mutation (*ERBB2*‐wt/amp: median 4.4 mutations per Mb, range: 1.8–16.7, *P* < 0.001, Fig. 1B). Chromosomal instability, inferred from the fraction of genome altered (FGA), was low in *ERBB2*‐non‐amp ECs, particularly those with *ERBB*2 mutation (*ERBB2*‐mut/non‐amp: 0.5%, range: 0–33.1% vs *ERBB2*‐wt/non‐amp: 5.1%, range: 0–95.7%, *P* < 0.001), in contrast to *ERBB2*‐wt/amp ECs, which typically showed high FGAs (23.4%, range: 0–71.0%, *P* < 0.001; Fig. 1C). Overall, the rare ECs with both *ERBB2* mutation and amplification (*ERBB2*‐mut/amp) had relatively low TMB (6.1 mutations per Mb, range: 3.3–14) and FGA (2.5%, range: 0.08–38.8%), though definitive conclusions cannot be drawn due to the limited numbers.

Assessment of cancer gene alterations revealed that the most frequent co‐existing mutations in *ERBB2*‐mut ECs involved *ARID1A* (65%), *PTEN* (57%) and *PIK3CA* (54%), which are characteristic of endometrioid and clear cell carcinomas [1, 24] (Fig. 1D). Genetic alterations typical of high‐grade ECs, namely, *TP53* (46%), *FBXW7* (35%) and *PPP2R1A* (17%), were also observed [1, 35].

For comparison, among the publicly available TCGA cohort of 529 ECs that underwent whole‐exome sequencing, 15 (2.8%) cases harbored *ERBB2* pathogenic mutations, of which one case harbored two distinct *ERBB2* mutations (V842I and L755S) [1]. Consistent with the results from our cohort, V842I (*n* = 4) and R678Q (*n* = 5) were the most common mutations, and the only other recurrent mutation was L755S (*n* = 3).

### 3.3 Clinicopathologic features and associations with molecular subtype

**Fig. 2:**
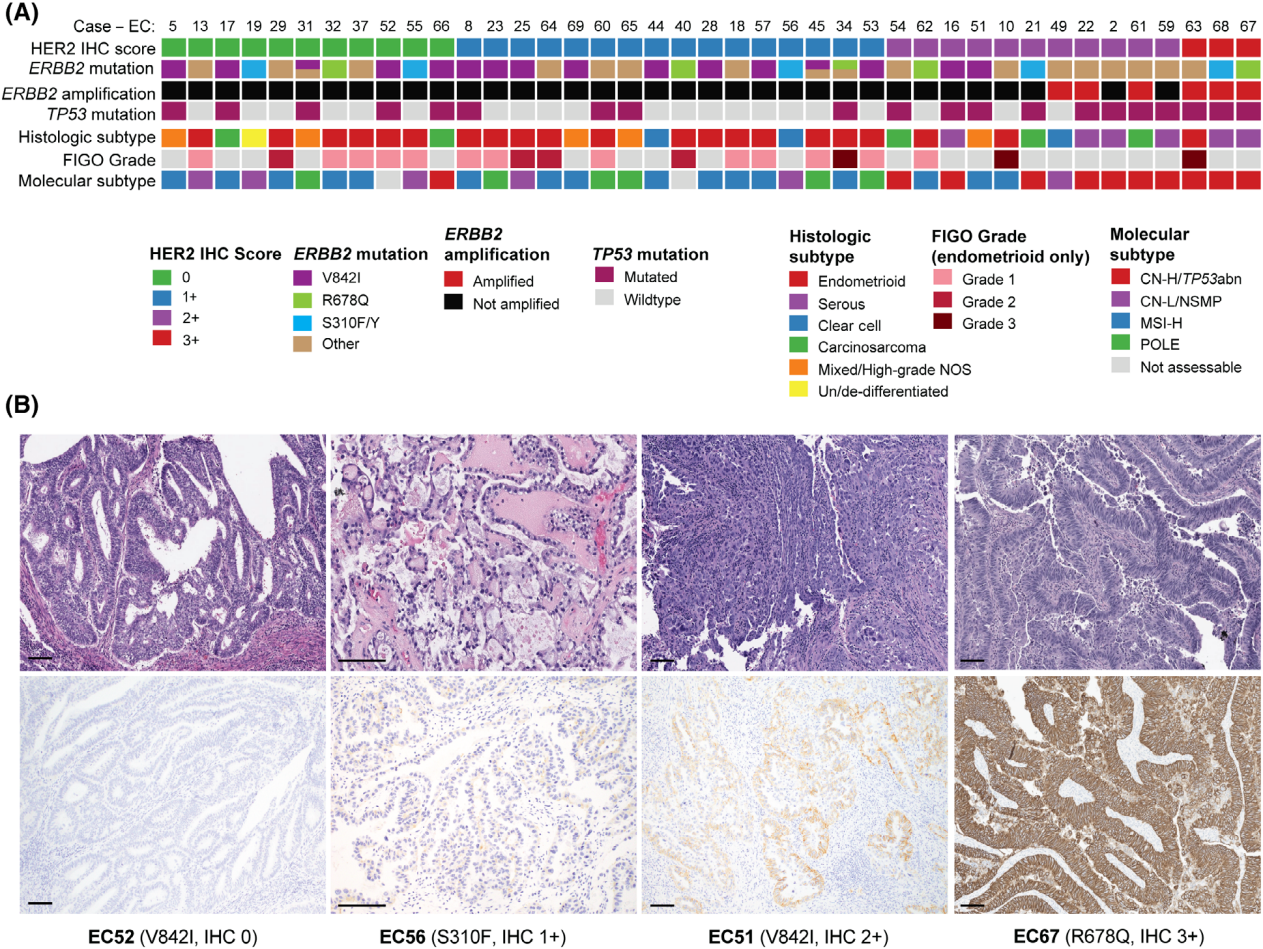
Histologic features and HER2 immunohistochemical analysis of *ERBB2*‐mutated endometrial carcinomas. (A) Stratification of *ERBB2*‐mutated endometrial carcinomas by HER2 immunohistochemistry (IHC) score (*n* = 41). HER2 IHC scores, and other tumor characteristics are color‐coded according to the legend. CN‐H/*TP53*abn, copy number‐high/*TP53* abnormal; CN‐L/NSMP, copy number‐low/no specific molecular profile; high‐grade EC‐NOS, high‐grade endometrial carcinoma, not otherwise specified; MSI‐H, microsatellite instability‐high. (B) Photomicrographs of representative cases (H&E and HER2 IHC): EC52, endometrioid carcinoma, Grade 1 (100× magnification); EC56, clear cell carcinoma (200× magnification); EC51, high‐grade EC‐NOS (100× magnification); EC67, serous carcinoma (*ERBB2*‐mutated and amplified, 100× magnification). Scale bar represents 100 μm.

The spectrum of EC histologic subtypes was represented among *ERBB2*‐mut/non‐amp ECs (Fig. 2A, Table 1), including endometrioid (66%; of which 79% were Grades 1 or 2), serous (6.9%) and clear cell (6.9%) carcinomas, carcinosarcoma (6.9%), mixed EC/high‐grade EC, not otherwise specified (10%), and undifferentiated/de‐differentiated EC (3.4%). Similar frequencies were observed across *ERBB2*‐wt/non‐amp ECs. However, significant differences became apparent when stratifying by molecular subtype [24]. Consistent with the high TMB observed in *ERBB2*‐mut/non‐amp ECs, these tumors were enriched for MSI‐H (59%) and *POLE* (11%) molecular subtypes (compared to 24% MSI‐H and 5.6% *POLE* in *ERBB2*‐wt/non‐amp ECs, *P* < 0.001). Of note, TMB was consistently higher in *ERBB2*‐mut/non‐amp compared to *ERBB2*‐wt/non‐amp ECs, even within MSI‐H (*ERBB2*‐mut/non‐amp: median 50.3 mutations per Mb, range: 4.4–88.6, vs *ERBB2*‐wt/non‐amp: median 29.8 mutations per Mb, range: 0.9–397.9; *P* < 0.001) and microsatellite‐stable (MSS; *ERBB2*‐mut/non‐amp: median 10.1 mutations per Mb, range: 3.3–436.2, vs *ERBB2*‐wt/non‐amp: median 5.3 mutations per Mb, range: 0.8–667.9; *P* < 0.001) subgroups, whilst no significant differences in FGA values were observed (Fig. S1A,B). Patients with *ERBB2*‐mut/non‐amp and *ERBB2*‐wt/non‐amp ECs had a similar age distribution (median 60 vs 63, *P* = 0.041) and did not significantly differ with respect to body mass index (BMI, median 28.0 vs 29.7 kg·m^−2^, *P* = 0.08) or stage at presentation (*P* = 0.22). Consistent with our findings, in the TCGA cohort [1], most *ERBB2*‐mut ECs (11/15, 73%) were endometrioid and of MSI‐H molecular subtype (*n* = 11, 73%).

**Table 1:** Demographic and clinicopathologic features of endometrial cancers stratified by *ERBB2* genetic alteration status. The number of cases in each category do not always sum up to the total number of cases in the cohort due to missing data values. amp, amplified; CN‐H/*TP53*abn, copy number‐high/*TP53* abnormal; CN‐L/NSMP, copy number‐low/no specific molecular profile; FIGO, International Federation of Gynecology and Obstetrics; High‐grade EC‐NOS, high‐grade endometrial carcinoma, not otherwise specified; MSI‐H, microsatellite instability‐high; mut, mutated; non‐amp, non‐amplified; wt, wildtype.

Characteristic	*ERBB2* mutation/copy number status, *N* = 1859				*P*‐value^a^	
	wt/non‐amp, *N* = 1691, *n* (%)	mut/non‐amp, *N* = 58, *n* (%)	wt/amp, *N* = 99, *n* (%)	mut/amp, *N* = 11, *n* (%)	mut/non‐amp vs wt/non‐amp	mut/non‐amp vs wt/amp
Age at diagnosis [median, years (range)]	63 (21–96)	60 (31–83)	66 (54–86)	70 (54–75)	0.041	< 0.001
BMI [median, kg·m^−2^, (range)]	29.7 (14.9–67.6)	28.0 (16.9–48.4)	28.3 (19.3–48.2)	29.3 (21.6–52.7)	0.08	0.95
Stage (FIGO 2009)						
I	906 (59)	40 (71)	25 (29)	3 (33)	0.22	< 0.001
II	65 (4.2)	3 (5.4)	4 (4.7)	1 (11)		
III	309 (20)	9 (16)	24 (28)	2 (22)		
IV	251 (16)	4 (7.1)	32 (38)	3 (33)		
Histologic type						
Endometrioid	948 (56)	38 (66)	7 (7.1)	1 (9.1)	0.12	< 0.001
Serous	233 (14)	4 (6.9)	34 (34)	8 (73)		
Clear cell	47 (2.8)	4 (6.9)	5 (5.1)	1 (9.1)		
Carcinosarcoma	196 (12)	4 (6.9)	25 (25)	1 (9.1)		
Mixed/high‐grade EC‐NOS	140 (8.3)	6 (10)	23 (23)	0 (0)		
Undifferentiated/de‐differentiated	36 (2.1)	2 (3.4)	1 (1.0)	0 (0)		
Unclassifiable	91 (5.4)	0 (0)	4 (4.0)	0 (0)		
FIGO grade (for endometrioid only)						
1 or 2	741 (81)	30 (79)	5 (71)	0 (0)	0.84	0.64
3	175 (19)	8 (21)	2 (29)	1 (100)		
Molecular subtype						
*POLE*	95 (5.6%)	6 (11%)	0	0	< 0.001	< 0.001
MSI‐H	404 (24%)	32 (59%)	0	0		
CN‐L/NSMP	561 (33%)	7 (13%)	8 (8.1%)	1 (9.1%)		
CN‐H/*TP53*abn	631 (37%)	9 (17%)	91 (92%)	10 (91%)		

a Fisher exact test, two‐tailed.

Significant differences between *ERBB2*‐mut/non‐amp versus *ERBB2*‐wt/amp ECs were observed with respect to age (*P* < 0.001), stage (*P* < 0.001), and distribution of histologic (*P* < 0.001) and molecular subtypes (*P* < 0.001; Table 1). Specifically, patients with ECs with *ERBB2* amplification were significantly older, more frequently presented at advanced stage, with enrichment of high‐grade histologic types (serous/mixed carcinomas and carcinosarcoma), and CN‐H/*TP53*abn molecular subtype. Of the 11 *ERBB2*‐mut/amp ECs, 10/11 were CN‐H/*TP53*abn (serous, *n* = 8, grade 3 endometrioid, *n* = 1, carcinosarcoma, *n* = 1), and the remaining case was an MSI‐H clear cell carcinoma.

### 3.4 Genetic features of MSI‐H ERBB2‐mutated ECs

Across *ERBB2*‐mut ECs, those of MSI‐H molecular subtype were particularly enriched for V842I and R678Q hotspot mutations (25/32, 78%, of MSI‐H, vs 14/33, 42%, of other molecular subtypes, *P* = 0.005). Of the 32 *ERBB2*‐mut EC of MSI‐H molecular subtype, the mechanism of MMR‐deficiency/MSI varied, with 9 (28%) being associated with *MLH1* promoter hypermethylation. Of those negative for *MLH1* promoter hypermethylation, available germline testing results revealed 9/21 (43%) cases were associated with Lynch syndrome, with an underlying pathogenic germline mutation in one of the MMR genes, including *MLH1* (*n* = 1), *MSH2* (*n* = 4) or *MSH6* (*n* = 4). In addition, 1 (4.8%) had an *MUTYH* germline mutation (along with *MSH2* somatic mutations). Somatic MMR gene mutations were present in 12/21 (57%) cases. For the two patients that were negative for *MLH1* promoter hypermethylation and of unknown germline status, one had isolated MSH6 loss by IHC without any MMR gene mutations, and the other had loss of MSH2 and MSH6 expression and co‐existing *MSH2* tumor mutations.

### 3.5 Immunohistochemical analysis of HER2 protein expression in ERBB2‐mutated ECs

HER2 IHC was performed (Fig. 2A,B) on 41 *ERBB2*‐mut ECs with available tissue and demonstrated that the majority had low levels of HER2 expression, with the following distribution of HER2 IHC scores: 0, 27% (*n* = 11); 1+, 39% (*n* = 16); 2+, 27% (*n* = 11); and 3+, 7.3% (*n* = 3). All tumors with IHC 3+ and 3/11 (27%) with IHC 2+ harbored both *ERBB2* mutation and amplification. There was a significant association between the presence of a *TP53* mutation and higher levels of HER2 protein expression, with *TP53* mutation observed in 11/14 (79%) ECs with HER2 IHC scores of 2+/3+, compared to 9/27 (33%) cases with HER2 IHC 0/1+ scores (*P* = 0.009). Similarly, the CN‐H/*TP53*abn molecular subtype (which excludes MSI‐H ECs with *TP53* mutation), was associated with increased HER2 protein expression (10/14, 71%, of HER2 IHC 2+/3+, vs 1/27, 3.7%, of HER2 IHC 0/1+, *P* < 0.001). The most common mutations (V842I, R678Q, S310F/Y) were observed in ECs across the range of HER2 IHC scores and there was no apparent relationship between specific mutation and HER2 expression level.

### 3.6 Clinical outcomes and response to trastuzumab therapy

**Fig. 3:**
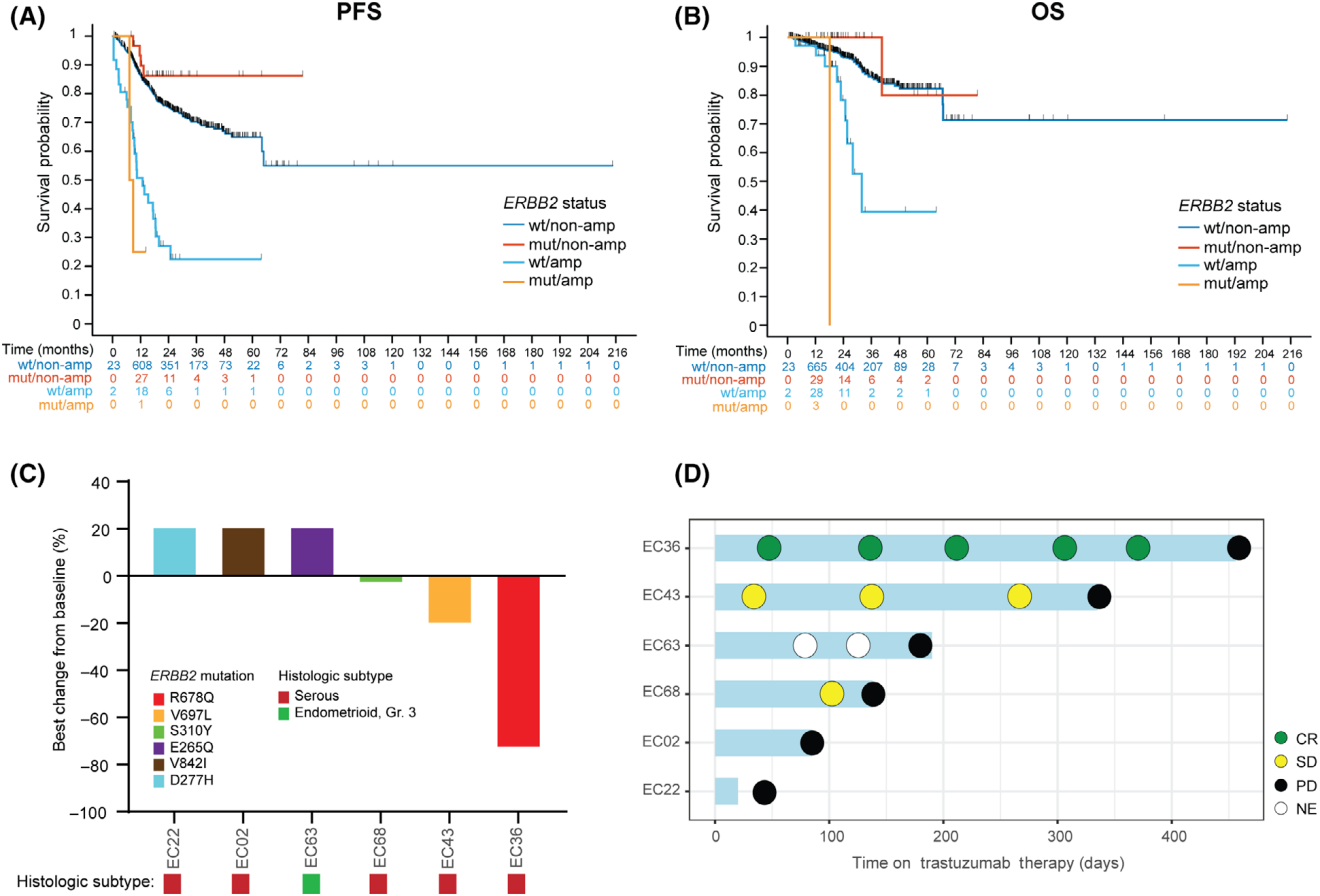
Clinical outcomes of *ERBB2*‐mutated endometrial carcinomas. (A) Progression‐free (PFS) and (B) overall survival (OS) in endometrial carcinomas stratified by *ERBB2* mutation (wt, wildtype; mut, mutated) and copy number (non‐amp, non‐amplified; amp, amplified) status. (C) Waterfall plot showing treatment responses to trastuzumab combined with chemotherapy. In EC02, EC22, and EC63, bars showing a 21% increase denotes appearance of new non‐target lesions at first evaluable computed tomography scan performed while on treatment. Specific *ERBB2* mutations and histologic subtype are color‐coded as indicated in the legend. (D) Swimmer's plot showing best response to trastuzumab, time on treatment, and time to disease progression. CR, complete response; NE, not evaluable (non‐CR, non‐PD); PD, progression of disease; PR, partial response.

The median follow‐up for the 1012 EC patients who met criteria for survival analysis (see Section 2; *ERBB2*‐wt/non‐amp, *n* = 936; *ERBB2*‐mut/non‐amp, *n* = 37; *ERBB2*‐wt/amp, *n* = 39; *ERBB2*‐mut/amp, *n* = 3) was 21.8 months (range 0.6–214.5 months) and there were 83 deaths. Median PFS was not reached for non‐amplified *ERBB2*‐wt and *ERBB2*‐mut ECs (mut/non‐amp vs wt/non‐amp: HR 0.51, 95% CI 0.19–1.36) and was 12.8 (95% CI 7.8–18.4) months for *ERBB2*‐wt/amp ECs (wt/amp vs wt/non‐amp: HR 4.32, 95% CI 2.84–6.57, *P* < 0.001; Fig. 3A, Table S1). Median OS was not reached for *ERBB2*‐wt/non‐amp and *ERBB2*‐mut/non‐amp ECs (mut/non‐amp vs wt/non‐amp: HR 0.39, 95% CI 0.05–2.78) and was 31.9 (95% CI 24.8‐NE) months for *ERBB2*‐wt/amp ECs (wt/amp vs wt/non‐amp: HR 4.35, 95% CI 2.16–8.76, *P* < 0.001; Fig. 3B). PFS and OS were un‐estimable for *ERBB2*‐mut/amp ECs, due to limited sample size. *ERBB2* genetic alteration status was no longer significant on multivariate analysis after adjusting for age, stage and molecular subtype (Table 2). Among *ERBB2*‐mut ECs, no survival differences were observed between cases harboring V842I or R678Q *ERBB2* mutations compared to other pathogenic *ERBB2* mutations.

**Table 2:** Multivariate analysis for progression‐free and overall survival. CI, confidence interval; CN‐H/*TP53abn*, copy number‐high/*TP53* abnormal; CN‐L/NSMP, copy number‐low/no specific molecular profile; FIGO, International Federation of Gynecology and Obstetrics; HR, hazard ratio; MSI‐H, microsatellite instability‐high.

Characteristic	Progression‐free survival		Overall survival	
	HR (95% CI)	*P*‐value	HR (95% CI)	*P*‐value
Age	1.02 (1.00–1.03)	0.029	1.04 (1.01–1.06)	0.006
Molecular subtype				
POLE/MSI‐H	–	< 0.001	–	< 0.001
CN‐L/NSMP	1.01 (0.64–1.59)		1.47 (0.64–3.38)	
CN‐H/*TP53*abn	2.86 (1.98–4.13)		3.91 (1.94–7.86)	
Stage (FIGO 2009)				
I/II	–	< 0.001	–	< 0.001
III	3.96 (2.82–5.55)		3.78 (2.07–6.90)	
IV	7.18 (5.09–10.1)		7.86 (4.52–13.7)	
*ERBB2* status				
Wildtype	–	0.52	–	0.69
Mutated	0.89 (0.33–2.43)		0.54 (0.07–3.89)	
Amplified	1.28 (0.83–1.99)		1.24 (0.60–2.56)	

Six patients with *ERBB2*‐mut ECs received anti‐HER2 therapy (Fig. 3C,D). All patients were treated with trastuzumab combined with chemotherapy in the recurrent setting. By MSK‐IMPACT, 5 also harbored concurrent *ERBB2* amplification; in the remaining case (EC22), low level *ERBB2* amplification was detected by fluorescence *in situ* hybridization only (*ERBB2/CEP17* ratio: 2.1, *ERBB2* copy number: 3.5). Trastuzumab was administered once every 3 weeks and number of doses received ranged from 1 to 19 (median 8), with treatment lasting until disease progression. Clinical responses with associated specific *ERBB2* mutations were as follows: complete response, *n* = 1 (R678Q), stable disease, *n* = 2 (V697L, S310Y), and progressive disease, *n* = 3 (V842I, D277H, E265Q). Median time from treatment initiation to disease progression was 164 days (range 20–456 days).

## 4 Discussion

Across epithelial malignancies, oncogenic activation of *ERBB2* occurs predominantly by gene amplification and less commonly by mutation. In EC, the prevalence of *ERBB2* amplification has been reported to be 3.8% across all histologic subtypes [9]. In the current study, we show that a comparable proportion of ECs (2.6%) harbor *ERBB2* mutations. While *ERBB2* amplification is essentially exclusive to high‐grade histologic types, including serous ECs and carcinosarcomas, of CN‐H/*TP53*‐altered molecular subtype [9, 24, 35], *ERBB2* mutations occur predominantly in an MSI‐H background, and most are low‐grade endometrioid carcinomas. Furthermore, while *ERBB2* amplification leads to protein overexpression, the majority of *ERBB2*‐mut ECs have low or undetectable levels of HER2 expression by IHC. Our results indicate that *ERBB2* mutations and amplification, although involving the same gene, define distinct pathologic subgroups of EC, and may necessitate distinct therapeutic approaches.

The observation that *ERBB2*‐mut ECs have high TMB and associated with MSI‐H and to a lesser extent, *POLE* molecular subgroups, suggests that *ERBB2* mutation originated as part of a “mutator phenotype.” *ERBB2* mutations have also been reported at higher frequency in MSI‐H compared to microsatellite‐stable colorectal cancers. In most of the EC cases, *ERBB2* mutations were clonal, however, indicating that they likely occurred early in carcinogenesis, followed by selective clonal expansion. Furthermore, our cohort was restricted to *ERBB2* variants that were annotated as mutational hotspots and/or “pathogenic.” Our analyses thus provide compelling evidence that *ERBB2* mutations are true pathogenic drivers in EC, rather than mere passenger mutations, even when occurring in the context of a high TMB/MSI‐H background.

Despite *MLH1* promoter hypermethylation being the more prevalent cause for MMR‐deficiency in EC (~ 70% of MMR‐deficient ECs) [36], MSI‐H ECs with *ERBB2* mutations were enriched for germline or somatic MMR mutations (up to 72%). These results complement previous work that reported *ERBB2* mutations in only 3% of ECs with *MLH1* promoter hypermethylation, compared to 29% and 13% of ECs with MMR germline and somatic mutations, respectively [36]. The biological explanation for this observation is unclear. However, ECs with *MLH1* promoter hypermethylation showed lower TMB compared to those harboring MMR germline/somatic mutations [36], which further suggests that *ERBB2* activation may selectively promote tumor cell survival in the setting of high TMB.

The prognostic impact of *ERBB2* mutations varies across other tumor types. In breast, *ERBB2* mutations are associated with poorer OS in invasive lobular, but not ductal, carcinomas [13]. Furthermore, higher rates of complete response to chemotherapy were achieved in *ERBB2*‐mut compared to *ERBB2*‐wt bladder cancers [14], while no associations with clinical outcomes were observed in lung cancer [15]. Similarly, *ERBB2* mutation status was not associated with prognosis in our EC cohort.

The V842I and V678Q hotspot mutations, in the kinase and juxtamembrane domains respectively, are by far, the most frequent pathogenic variants in EC, together making up 61% of cases, followed by S310F/Y (8.7%). Interestingly, these are also the 3 most common *ERBB2* mutations observed in colorectal cancer [37]. Future work is necessary to determine whether these specific mutations preferentially drive carcinogenesis in intestinal and endometrial epithelial cells, particularly in the context of an MSI‐H/high mutation burden genetic background.

Pre‐clinical studies have shown variability with respect to kinase activity, phosphorylation of downstream signaling proteins, transformation potential and drug sensitivity between different mutations [11, 16, 38, 39]. Concerning the most prevalent mutations in our EC cohort, V842I confers *in vitro* resistance to trastuzumab and a reversible kinase inhibitor, lapatinib, while conflicting data to neratinib was reported across studies [11, 16, 40]. In contrast, R678Q is associated with sensitivity to trastuzumab, lapatinib and neratinib [38, 39]. There is more evidence supporting S310F/Y to be sensitive to anti‐HER2 therapy. A patient‐derived xenograft model of S310Y‐mutated colorectal cancer was sensitive to trastuzumab, lapatinib and neratinib, with the highest activity observed in trastuzumab combined with neratinib [40]. Cabel et al. [41] reported two patients, one with cervical and the other with EC, both harboring *ERBB2* S310Y mutation, who achieved partial responses after treatment with a combination regimen of paclitaxel, trastuzumab, and everolimus (due to co‐existing mTOR pathway alteration). In the SUMMIT trial, 3 of 12 patients with cervical cancer treated with neratinib achieved partial responses, and all 3 had tumors with S310Y/F mutations, and one had a V842I mutation [18, 42].

In our cohort, only six patients received trastuzumab therapy, due to concurrent HER2 overexpression/amplification. Interestingly, the only two patients with objective responses had tumors with *ERBB2* mutations at positions R678Q and S310Y, respectively, whilst a patient with an *ERBB2*‐V842I‐mutated EC progressed on therapy, consistent with pre‐clinical functional characterization of these mutations. Co‐existing *ERBB2* mutation and amplification is rare and observed in < 5% of solid tumors [43]. Given that most *ERBB2* mutations, particularly those in the kinase domain, are associated with resistance to trastuzumab, prior work has shown that in *ERBB2*‐amp metastatic breast cancer patients who received trastuzumab combined with chemotherapy as first‐line treatment, those with concurrent *ERBB2* mutations had shorter PFS compared to the *ERBB2*‐wt group (median PFS 4.7 vs 11.0 months) [44].

In the SUMMIT basket trial, of the seven patients with *ERBB2*‐mut EC treated with neratinib, the best response was stable disease in four patients, with *ERBB2* mutations at S310Y (*n* = 2), V777L (*n* = 1) and R678Q (*n* = 1), and disease progression in three patients, with *ERBB2* mutations at V842I, V697L, and P761del [18]. While the numbers are small, neratinib alone does not appear to be particularly effective for *ERBB2*‐mut EC. Combination therapy with other therapeutic agents or alternative HER2‐directed therapies should be explored for this patient population. Given the association between *ERBB2*‐mutation and MSI‐H status, many of these patients would be eligible for immunotherapy [45]; hence combining anti‐HER2 therapy with an immune checkpoint inhibitor may represent a potential strategy.

Emerging HER2 ADCs, which have demonstrated clinical efficacy in HER2‐low/negative tumors may be particularly promising for *ERBB2*‐mut ECs, most of which, have low or undetectable HER2 expression by IHC. Recent work has demonstrated that *ERBB2* mutations enhance internalization of receptor‐bound trastuzumab emtansine, and objective responses trastuzumab emtansine were observed in patients with *ERBB2*‐mut lung cancer patients, including those with low or undetectable (IHC score 0/1+) HER2 expression [19, 20]. Trastuzumab deruxtecan is a next‐generation HER2‐targeting ADC, which has previously demonstrated remarkable efficacy in HER2‐low breast cancer, attributed, to its potent cytotoxic payload with high drug‐to‐antibody ratio (8 : 1) and its bystander killing effect of neighboring HER2‐non‐expressing tumor cells [46]. In a Phase 2 trial of trastuzumab deruxtecan in patients with *ERBB2*‐mut lung cancer, most of whom lacked co‐existing *ERBB2* amplification, the objective response rate was 55% and durable responses were observed independent of HER2 expression level [21]. As the mechanism of action involves internalization of the receptor‐ADC complex to deliver the cytotoxic payload, rather than inhibition of downstream signaling, ADCs are efficacious across *ERBB2* mutations, involving extracellular or kinase domains.

The present study has some limitations inherent to its retrospective nature. The number of *ERBB2*‐mut ECs that were appropriate to be included in the survival analysis was small, being restricted to those who received their entire treatment course and follow‐up at our institution. Only 6 patients received trastuzumab therapy and all had *ERBB2* amplification/HER2 overexpression; with this small sample size, definitive conclusions cannot be drawn concerning the impact of specific mutations on treatment response. Nevertheless, consistent with previous reports [18, 38, 39, 40, 41], clinical benefit was observed in the ECs with *ERBB2*‐R678Q and S310Y mutations, while the *ERBB2*‐V842I‐mutated EC was among those resistant to treatment.

## 5 Conclusions

This retrospective cohort study characterizes the clinicopathologic features and molecular genetic landscape of ECs harboring pathogenic *ERBB2* mutations, thereby defining a rare subgroup of ECs, which is enriched for MSI‐H molecular subtype and pathogenically distinct from *ERBB2*‐wt and *ERBB2*‐amp ECs. Future prospective trials will be needed to assess the efficacy of other HER2 targeting agents in *ERBB2*‐mut EC, either as monotherapy or part of a combination regimen, which may include immunotherapy. Since *ERBB2*‐mut and *ERBB2*‐amp ECs constitute largely non‐overlapping groups, our results suggest that more ECs patients may potentially benefit from novel anti‐HER2 therapies.

## Conflict of interest

BW reports grant funding by Repare Therapeutics, outside the scope of the current study. The remaining authors declare no competing interests.

## Author contributions

BW and MHC contributed to conception, design, and supervision. PS contributed to bioinformatics analysis. QZ and AI contributed to statistical analysis. MNB, PS, SM, WM, CD, TB, NRA‐R, CA, LHE, BW, and MHC contributed to data collection. MNB, MHC, and BW contributed to data interpretation/analysis. All authors reviewed, edited, and approved the manuscript.

### Peer review

The peer review history for this article is available at https://www.webofscience.com/api/gateway/wos/peer‐review/10.1002/1878‐0261.13698.

## Supporting information


**Fig. S1.** *ERBB2*‐mutated endometrial carcinomas are associated with increased tumor mutational burden.
**Table S1.** Univariate associations with progression‐free and overall survival.

## Data Availability

Targeted sequencing data supporting the findings of this study will be available at cBioPortal for Cancer Genomics (www.cbioportal.org) upon publication of this manuscript.
