# Supplementary material for: ERBB2 mutations define a subgroup of endometrial carcinomas associated with high tumor mutational burden and the microsatellite instability‐high (MSI‐H) molecular subtype
Source: Mol Oncol. 2024 Jul 19;18(10):2356–68. doi: 10.1002/1878-0261.13698 (PMC11459037; doi:10.1002/1878-0261.13698)
Supplement: Supplementary file 1 — Fig. S1. ERBB2‐mutated endometrial carcinomas are associated with increased tumor mutational burden. Table S1. Univariate associations with progression‐free and overall survival. [file MOL2-18-2356-s001.docx]

**SUPPLEMENTARY INFORMATION**

***ERBB2* mutations define a subgroup of endometrial carcinomas associated with high tumor mutational burden and the microsatellite instability-high (MSI-H) molecular subtype**

Brodeur MN, Selenica P, Ma W, Moufarrij S, Dagher C, Basili T, Abu-Rustum NR, Aghajanian C, Zhou Q, Iasonos A, Ellenson LH, Weigelt B, Chui MH

**
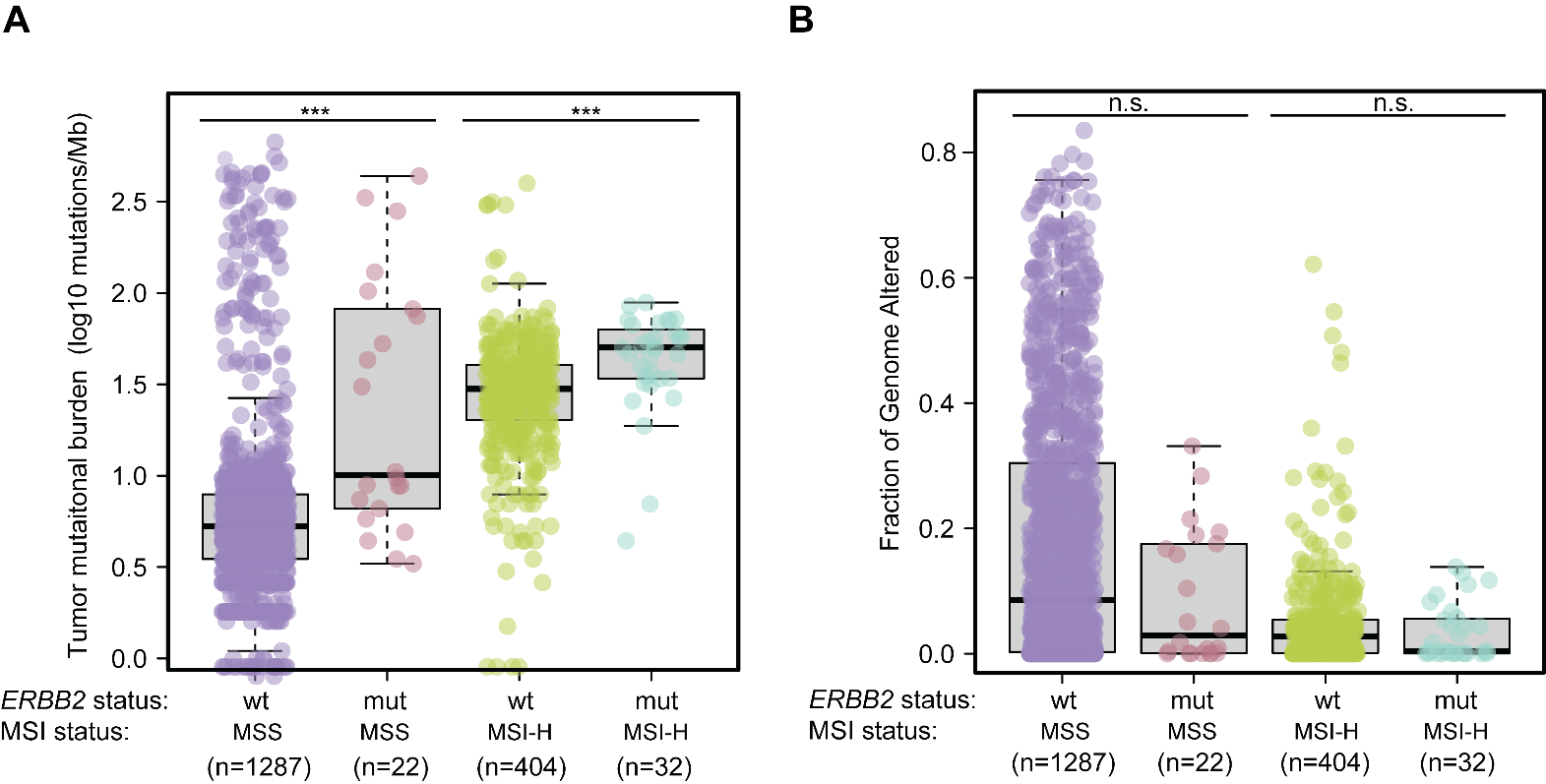
**

**Supplementary Figure S1.** *ERBB2*-mutated endometrial carcinomas are associated with increased tumor mutational burden. **(A)** Tumor mutational burden and **(B)** fraction of genome altered, in endometrial carcinomas stratified by *ERBB2* mutational status and microsatellite instability (MSI) status. Only *ERBB2*-non-amplified cases are included. wt – wildtype, mut – mutated, MSS – microsatellite stable, MSI-H – microsatellite instability-high. *** p<0.001.

**Supplementary Table S1: Univariate associations with progression-free and overall survival**

| **Characteristic** | **Progression-free survival** | | **Overall survival** | |
| --- | --- | --- | --- | --- |
|  | HR (95% CI) | p-value | HR (95% CI) | p-value |
| **Age** | 1.03 (1.02-1.04) | <0.001 | 1.05 (1.03-1.07) | <0.001 |
| **Molecular subtype** |  |  |  |  |
| POLE/MSI-H | - | <0.001 | - | <0.001 |
| CN-L/NSMP | 0.91 (0.58-1.41) |  | 1.42 (0.62-3.25) |  |
| CN-H/*TP53*abn | 4.89 (3.48-6.89) |  | 7.18 (3.67-14.03) |  |
| **Stage (FIGO 2009)** |  |  |  |  |
| I/II | - | <0.001 | - | <0.001 |
| III | 4.62 (3.33-6.41) |  | 4.08 (2.27-7.32) |  |
| IV | 11.67 (8.52-15.99) |  | 11.52 (6.81-19.51) |  |
| ***ERBB2* status** |  |  |  |  |
| Wildtype | - | <0.001 | - | <0.001 |
| Mutated | 0.51 (0.19-1.36) |  | 0.39 (0.05-2.78) |  |
| Amplified | 4.32 (2.84-6.57) |  | 4.35 (2.16-8.76) |  |

HR – hazard ratio; CI – confidence interval; FIGO – International Federation of Gynecology and Obstetrics; CN-L/NSMP – copy number-low/no specific molecular profile; CN-H/*TP53*abn – copy number-high/*TP53* abnormal, MSI-H – microsatellite instability-high.
